# Uncovering the Roles of Septins in Cilia

**DOI:** 10.3389/fcell.2017.00036

**Published:** 2017-04-06

**Authors:** Oliva Palander, Maha El-Zeiry, William S. Trimble

**Affiliations:** ^1^Cell Biology Program, Hospital for Sick ChildrenToronto, ON, Canada; ^2^Department of Biochemistry, University of TorontoToronto, ON, Canada; ^3^Department of Physiology, University of TorontoToronto, ON, Canada

**Keywords:** septin, cilia, flagella, diffusion barrier, ciliogenesis

## Abstract

Septins are a family of GTP-binding proteins that associate with cellular membranes and the cytoskeleton. Their ability to polymerize into filamentous structures permits them to serve as diffusion barriers for membrane proteins and as multi-molecular scaffolds that recruit components of signaling pathways. At the cellular level, septins contribute to the regulation of numerous processes, including cytokinesis, cell polarity, cell migration, and many others. In this review, we discuss emerging evidence for roles of mammalian septins in the biogenesis and function of flagella and cilia, and how this may impact human diseases such as ciliopathies.

## Introduction

Septins are a family of poorly-characterized filamentous GTP-binding components of the cytoskeleton that orchestrate a variety of cellular processes including cytokinesis, cell migration, cell polarity, and cell-pathogen interactions (Fung et al., 2014). They were initially discovered in yeast in 1971 (Hartwell, 1971), and have since been identified in many eukaryotes (Onishi and Pringle, 2016). The number of septin genes varies widely amongst species, for example *Caenorhabditis elegans* has only two (UNC-59 and UNC-61) while there are thirteen in humans (SEPT1–SEPT12, SEPT14). In mammals, this complexity is further increased by use of alternative promoters and splicing, producing many different isoforms (for example see McIlhatton et al., 2001), making it challenging to fully understand the cell and molecular biology of these proteins.

Structurally, all mammalian septins contain a central GTP-binding domain, have variable N and C-termini, and the C-termini have different numbers of coiled-coil domains. Based on these features and sequence similarities, mammalian septin genes can be categorized into four subgroups (SEPT1, 2, 4, 5; SEPT6, 8, 10, 11, 14; SEPT7; SEPT3, 9, 12). Most cell types express at least one member of each group and these proteins assemble into ordered apolar complexes (Fung et al., 2014).

At the cellular level, septins are often found in structures with micron-scale membrane curvature (Bridges et al., 2016) such as the cytokinetic furrow in dividing cells (Maddox et al., 2007; Estey et al., 2010), the base of dendritic spines in neurons (Tada et al., 2007), the base of phagocytic cups (Huang et al., 2008), the annulus of sperm tails (Kwitny et al., 2010) and the base of primary cilia (Hu et al., 2010). In the following sections, we summarize what is known about septin function in general, discuss the evidence for their presence in flagella and different types of cilia, and speculate about their possible roles.

## Known functions of septins

The involvement of septins in myriad cellular processes indicates that they may have more than one function. Perhaps the best-studied example of multi-functionality occurs in yeast cytokinesis where septins form a ring from which the bud emerges (Longtine and Bi, 2003). Here, their filamentous nature allows them to function as a macromolecular scaffold to facilitate protein-protein interactions, where they recruit nearly 100 proteins, including bud-specific machinery and regulators of the actin cytoskeleton to the bud site (Gladfelter et al., 2001). This septin ring also acts as a diffusion barrier, limiting the lateral diffusion of membrane proteins between the mother and the bud (Takizawa et al., 2000).

Septins also bind to membranes, particularly to negatively charged lipids such as PIP2 (Zhang et al., 1999). This ability appears to also be influenced by the curvature of the membrane itself (Bridges et al., 2016) suggesting septins preferentially assemble on membrane surfaces of defined shape and charge. The binding of septins to membranes has been shown to promote membrane tubulation *in vitro* (Tanaka-Takiguchi et al., 2009), promote the retraction of blebs from the cell membrane (Gilden et al., 2012) and stabilize the plasma membrane to allow directed cell migration in T cells (Tooley et al., 2009).

Other functions ascribed to septins include their ability to affect the organization and dynamics of other cytoskeletal elements. For example, Drosophila septins were shown to bundle and curve actin filaments (Mavrakis et al., 2014). Septins have also been implicated in altering microtubule dynamics by binding to, and inhibiting the activity of, the microtubule stabilizing protein MAP4 (Kremer et al., 2005). Interaction of microtubules with septins appears to influence microtubule growth by suppressing catastrophe at growing plus ends (Bowen et al., 2011) causing microtubules to track along septin filaments.

## Flagella and cilia

**Figure 1:**
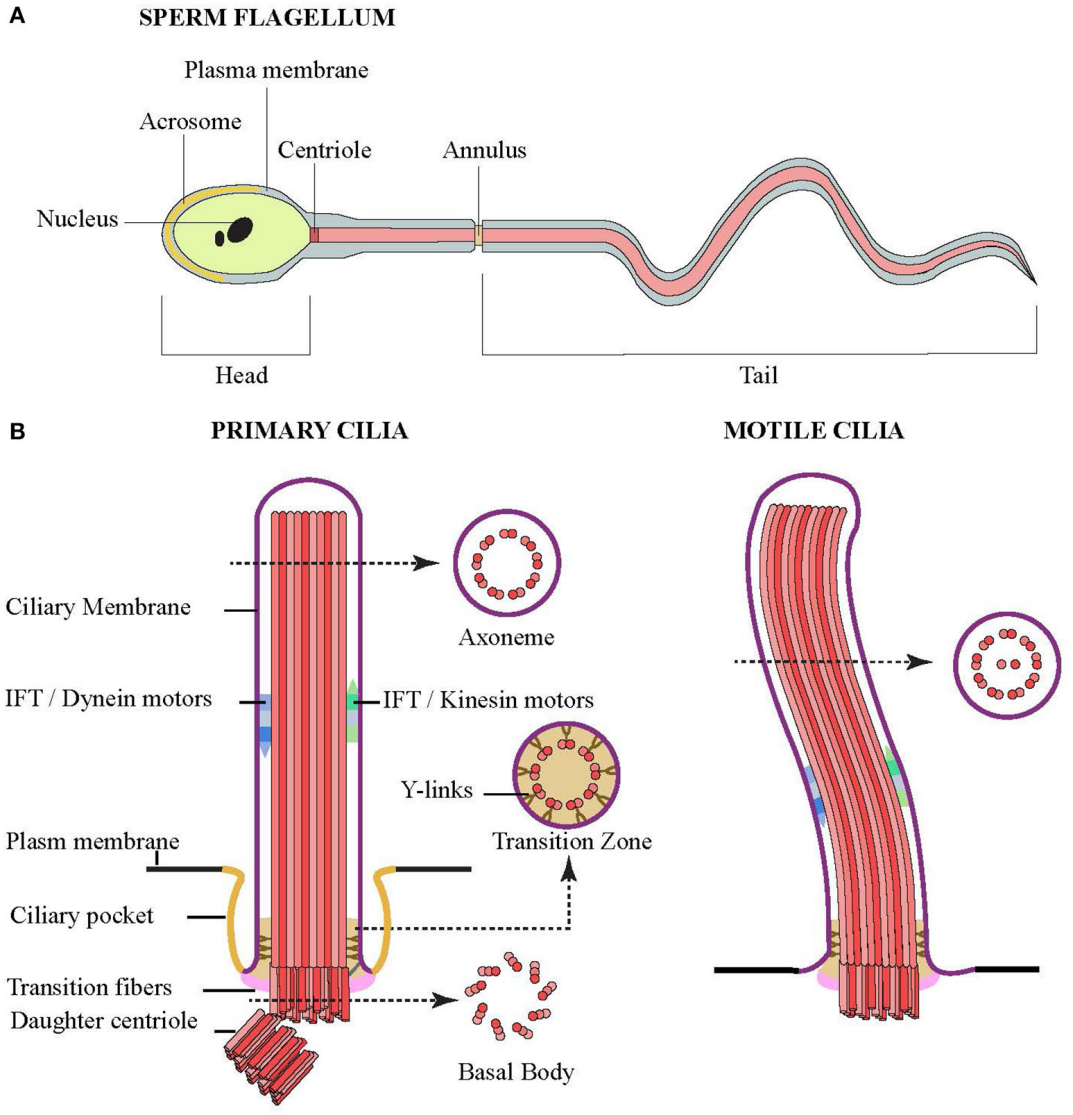
**Structures of flagella and cilia. (A)** Schematic diagram showing the structure of sperm composed of head (and nucleus), middle piece (centriole and annulus) and tail. The middle piece and tail are the prominent structure of flagellum that compose of a core of microtubules, whose movement is powered by flagellar dynein. **(B)** Schematic side view of primary (left) and motile (right) cilia. Cross sections showing microtubule organization of the axoneme, transition zone and basal body are indicated by arrow.

In vertebrates there are two types of cilia: (a) motile cilia, which are structurally identical to flagella, and (b) non-motile cilia, also called primary cilia. Diseases resulting from defects in cilia are collectively called ciliopathies, although the nature of the disease depends on the type of cilia affected. Flagella and cilia are microtubule-based organelles protruding from the cell surface. The term “flagellum” is used when a single motile cilium is used by cells for locomotion (for example, on mammalian sperm; Figure 1A). Motile cilia are more frequently found in large clusters on the cell surface and are involved in moving the extracellular fluid, rather than moving the cell itself. They are found lining the airway tract ependyma in the brain and the oviducts. Defects in motile cilia lead to ciliary dyskinesia and sterility. In contrast is the single motile primary cilia covering the node of the vertebral embryos, where a single cilium per cell moves in a circular manner to create the left-right asymmetry essential for correct positioning of visceral organs in the developing embryo (Baker and Beales, 2009). Defects in nodal cilia result in situs inversus, or loss of the typical asymmetry of the organs.

Rather than motile cilia, most cells have a single “primary” immotile cilium protruding from the membrane. Once thought to be extraneous structures (Webber and Lee, 1975), they are now considered important sensory organelles that act as cellular antennas to transmit extracellular cues into the cell. They are sites for the regulation of several developmental signaling pathways such as non-canonical Wnt and Sonic hedgehog pathways (Sasai and Briscoe, 2012). Diseases associated with loss of primary cilia include Meckel–Gruber Syndrome, Bardet–Biedl syndrome, Joubert syndrome and Polycystic kidney disease. These multisystem disorders frequently include retinal degeneration and cyst formation in liver and kidneys but interestingly may also include situs inversus, suggesting that genetic alterations resulting in the expression of dysfunctional ciliary proteins may affect more than one type of cilium (Baker and Beales, 2009).

In part, this is likely due to the fact that the structure of cilia is conserved across different cell types and species. When viewed in cross-section, cilia can be divided into three regions: basal body, transition zone, and axoneme (Figure 1B). The basal body is the region at the base of the cilium, which bears the centriole from which the ciliary machinery arises. Here, the 9 triplet microtubules of the mother centriole are attached to the periciliary membrane by transition fibers. The body of the cilium, or axoneme, follows and contains 9 doublet microtubules. In motile cilia, the microtubules are arranged in a “9+2” arrangement where the 9 doublets surround a central pair of singlet microtubules. In contrast, immotile primary cilia lack the central pair with their “9+0” arrangement.

In both the motile and primary cilia, the proximal region of the axoneme is known as the transition zone, and was shown to contain Y-shaped structures linking the microtubules doublets to the ciliary membrane (Reiter et al., 2012). This region is thought to provide some sort of gating to control the movement of proteins and lipids in and out of the cilia, which would explain the unique profile in both the ciliary membrane and lumen. Most but not all primary cilia, and some motile cilia, also have an endocytic membrane domain called the ciliary pocket near the base of the cilium (Ghossoub et al., 2011).

Intriguingly, several zebrafish studies have indicated the importance of septins for the proper formation and function of cilia, where loss-of-function studies have generated several phenotypes that resemble human ciliopathies (Dash et al., 2014). Hence, an understanding of the role of septins in ciliogenesis at a cellular level is critical to our understanding of ciliopathies.

## Localization of septins in cilia

Often the subcellular distribution of a protein can give insights into its function, so understanding septin localization in cilia is critical. Surprisingly, however, even though septins are involved in ciliogenesis in a variety of cell types and organisms, reports vary regarding their location within cilia.

**Figure 2:**
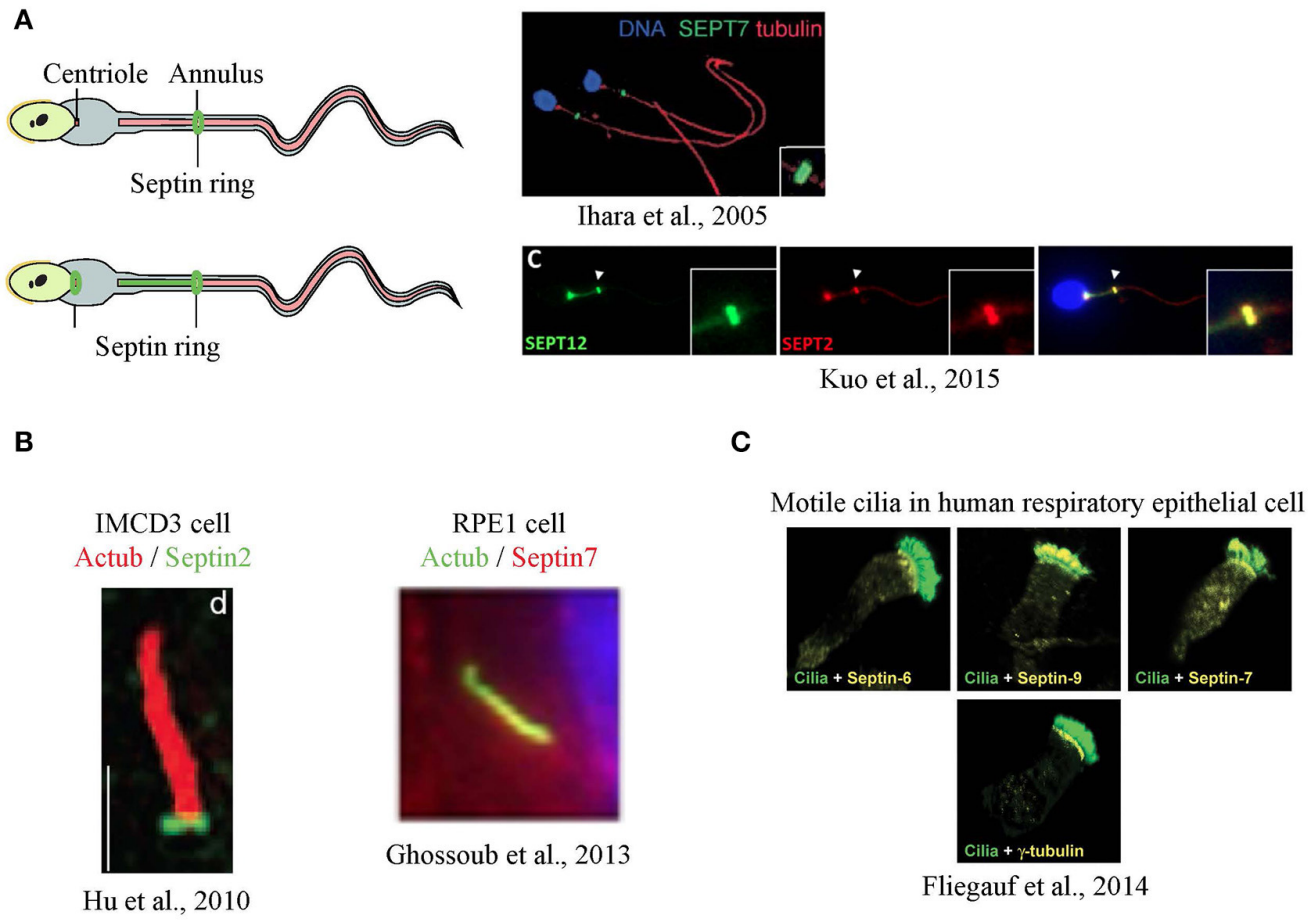
**Localization of septins in flagella and cilia. (A)** Septins form a ring at the annulus of mouse sperm (Ihara et al., 2005), and a secondary signal was also at the centriole in humans (Kuo et al., 2015) **(B)** In primary cilia, septins were detected as a ring at transition zone (Hu et al., 2010) or along the axoneme (Ghossoub et al., 2013) **(C)** In airway epithelia, different septins are detected at the base, along the axoneme or both in motile cilia (Fliegauf et al., 2014). Images in this figure are reproduced with permission from the relevant publishers.

Septins were first seen in a cilium-related structure when they were localized to the annulus of sperm flagella, a structure that has been suggested to separate the membrane compartments of the posterior and anterior tail regions (Cesario et al., 1995; Ihara et al., 2005; Kissel et al., 2005; Steels et al., 2007). Sperm from SEPT4-null mice exhibit a defective morphology where a clear annulus is missing, resulting in a fragile segment that causes the sperm's tail to have sharp bends, rendering the sperm immotile. A defective sperm annulus and the bent tail phenotype are also seen in sperm from infertile men with SEPT12 mutations and in SEPT12-mutant mice, confirming the importance of septins in sperm structural integrity and motility. In sperm from the SEPT4-null mice, basigin, a membrane protein that moves freely but is usually confined to anterior region of the tail, was found distributed all over the tail's plasma membrane, indicating that the compartmentalizing forces had been lost with the loss of the septin ring (Kwitny et al., 2010). The ring-like distribution is not always the only septin pattern in sperm, as later studies using human sperm showed septins, such as SEPT12, SEPT6 and SEPT2, localized from the centriole to the annulus (Kuo et al., 2015; Figure 2A).

The idea that septins might form a ring-like diffusion barrier in cilia was supported by the observation that SEPT2 could be seen as a ring at the base the primary cilia of mammalian cells (Hu et al., 2010) although some signal was reported along the axoneme (Figure 2B). In IMCD3 mouse kidney cells, the SEPT2 ring was localized to the transition zone and depletion of SEPT2 resulted in a significant shortening of the cilia. To test the function of SEPT2 at the transition zone, Nelson and colleagues designed GFP fusions of several ciliary transmembrane proteins, including serotonin receptor 6, Smoothened, and fibrocystin, and measured their fluorescence recovery after photobleaching (FRAP). Little exchange of these proteins between ciliary and non-ciliary pools was observed in control cells, despite both pools being mobile (Hu et al., 2010). However, the short cilia that formed upon depleting SEPT2 showed an increased entry of ciliary transmembrane proteins suggesting loss of a diffusion barrier.

Similarly, SEPT2 and SEPT7 had a ring-like appearance at the base of motile cilia in *Xenopus* embryos, and knockdown resulted in fewer and shorter cilia (Kim et al., 2010). Septin localization at the base of motile cilia was controlled by Fritz, a protein involved in organizing polarized cilia growth (Collier et al., 2005). Polymorphisms in Fritz were found in patients of Bardet-Biedl and Meckel-Gruber syndromes, suggesting a possible link between septin regulation and certain ciliopathies (Kim et al., 2010). It should be noted that SEPT7 was also present along the axoneme and at the basal body of these cilia.

If a septin ring at the base of cilia formed the diffusion barrier, and this barrier was needed for proper ciliogenesis, then septins should always be present as rings at the base of cilia. However, septins have more consistently been localized along the axoneme of cilia in many cell types and animal models (Ghossoub et al., 2013; Dash et al., 2014; Fliegauf et al., 2014; Zhai et al., 2014). For example, using various human cells lines such as RPE1 (human retinal pigment epithelial cell line) and hFE (human inner foreskin), Benmerah and colleagues found SEPT2, SEPT7 and SEPT9 to be co-localized along the axoneme in primary cilia and to regulate ciliary length, yet no rings were reported. Transiently expressed fluorescent septin fusion proteins also localized to the axoneme and FRAP revealed that axonemal SEPT2 was not exchanged with cytoplasmic septins, raising the possibility that it might be a structural component of the axoneme. In addition, they observed similar axonemal staining *in vivo* including in human kidney tubular cells (Ghossoub et al., 2013). This axonemal distribution is also supported by evidence from proteomic and proximity ligation experiments (Ishikawa et al., 2012; Mick et al., 2015).

While not always located at sites where diffusion barriers would be predicted to exist, septins could be involved in diffusion barrier formation. Indeed, Peterson and colleagues identified a protein complex containing 9 proteins (including B9D1 and TMEM231) that localized to the transition zone of primary cilia (Chih et al., 2012). Generation of knockout mice lacking either B9D1 or TMEM231 caused increased diffusion into the cilium and impaired hedgehog signaling, suggesting that these proteins comprised the diffusion barrier. Interestingly, depletion of SEPT2 inhibited assembly and recruitment of the complex to the transition zone.

Surprisingly, recent studies have shown that different septins had distinct localizations in motile cilia of human respiratory epithelial cells (Fliegauf et al., 2014). Some septins were found both at the ciliary base and the axoneme (SEPT2, 7, 9), while some were only at the base (SEPT6, 8) or the axoneme (SEPT11) (Figure 2C). This raises the possibility that distinct septin complexes could carry out different functions within cilia in the same cell.

In summary, the differential distribution of septins could arise in several ways. Septins at the base may accumulate there because they sense the micron-scale curvature of the ciliary membrane at this site (Bridges et al., 2016). Alternatively, the formation of ring-like structures of septins at the transition zone could be due to the concentration of specific lipids (Vieira et al., 2006) and/or other proteins at the base of cilia. With respect to septins in the axoneme of motile and primary cilia in various human cells, septins may play a structural role in the axonemal matrix (Ghossoub et al., 2013; Fliegauf et al., 2014). Since FRAP and live imaging of RPE1 cells showed no exchange between axonemal and cytoplasmic pools of septins, nor movement of septins within the axoneme, septins are not components of the intraflagellar transport system that moves proteins within cilia. However, septin accumulation in the axoneme could be due to the fact that septins directly bind microtubules (Bai et al., 2013) and the microtubules within the cilium are very stable. It is also important to consider that the apparently contradictory distributions of septins in cilia could be due to technical factors such cell type, specificity of antibody, fixation conditions, and maturation stage of the cilia. This could particularly be the case for staining at the base since many antibodies falsely stain centrosomes/basal bodies.

## What could be the function of septins in primary cilia?

The first function proposed for septins in cilia was as a diffusion barrier. Although septins are not always located as a ring at the base of cilia, several studies showed a loss of restricted diffusion into the remnant cilia following septin loss. In addition, loss of Hedgehog signaling following knockdown of septins was observed in Xenopus, Zebrafish and mammalian cells (Hu et al., 2010; Kim et al., 2010; Zhai et al., 2014). However, several cautions are required in considering these results. First, Hedgehog signaling depends on the presence and proper length of cilia, and since these have been impacted by loss of septins, so too may Hedgehog signaling. Second, the base of cilia has been shown to have a unique lipid composition (Vieira et al., 2006) that may itself contribute to restricted diffusion. It would be important to know if the absence of septins alters this lipid domain. Third, the remnant cilia that form in the absence of septins may be abnormal in many contexts including improper targeting of receptors to cilia or altered endocytosis. Changes in trafficking or recycling could affect the distribution of receptors in ways that resemble, but are distinct from, a simple passive restriction of lateral diffusion in the plane of the membrane (Trimble and Grinstein, 2015).

Depletion of septins consistently results in the loss or shortening of cilia and loss of ciliary function. A major consideration for all of these types of studies is whether the effect is due to the function of the septin directly at the cilium, or indirectly through one of its many effects on signaling pathways, membrane traffic or the cytoskeleton. For example, soluble tubulin levels are known to affect cilia length—more free tubulin results in longer cilia while less free tubulin leads to shorter cilia (Sharma et al., 2011). Septins have been shown to alter tubulin dynamics such that depletion of septins increases the numbers of stable (acetylated) microtubules (Kremer et al., 2005). This increased microtubule stabilization presumably reduces free tubulin levels globally in the cell, which could therefore be responsible for reduced cilia length. Similarly, since septins have widely accepted roles as scaffolding complexes, loss of signaling events normally occurring on septin filaments could impact signals required for cilia elongation. For example, the cilia and basal bodies of the SEPT7b morphants were also disoriented and resembled mutants in intraflagellar transport proteins (Jones et al., 2008; Cao et al., 2010). In Xenopus embryos, SEPT7 functions together with the planar cell polarity (PCP) protein Fritz to regulate ciliogenesis (Kim et al., 2010). Therefore, it is possible the role of SEPT7 in ciliogenesis is linked to the PCP pathway via Fritz (Park et al., 2015).

The function of PCP pathway in ciliogenesis is regulated by the exocyst complex, where the PCP protein Disheveled mediates recruitment of a sec8-positive vesicle to the basal body (Park et al., 2008). Septins also interact and regulate the exocyst complex by directing it to the correct location and affect the activity of SNARE proteins important in membrane fusion (Beites et al., 1999, 2005; Amin et al., 2008; Estey et al., 2010), suggesting that changes in membrane traffic caused by septins could also impact cilia growth and function. As well, SEPT7 was shown to interact with Rab8 (Dash et al., 2014), a small GTPase that functions in vesicle trafficking and ciliogenesis (Nachury et al., 2007; Yoshimura et al., 2007). Septins could also affect vesicle traffic via the cytoskeleton since they associate with microtubules and guide the direction of microtubule growth (Bowen et al., 2011; Nölke et al., 2016) and may control vesicular transport along them (Spiliotis et al., 2008).

## Conclusions

In sum, despite ample evidence linking septin function to ciliogenesis, there remain more questions than answers about their roles in this process. Since septin depletion *in vivo* gives phenotypes implicating nodal, motile and primary cilia, the function of septins in ciliogenesis likely involves something conserved among all cilia. Future work will be needed to decipher the contributions of septins to ciliogenesis, and to examine their possible contribution to ciliopathies.

## Author contributions

OP, ME, and WT wrote the manuscript. OP and ME contributed equally to this work.

### Conflict of interest statement

The authors declare that the research was conducted in the absence of any commercial or financial relationships that could be construed as a potential conflict of interest.
